# Do shoulder kinematics change after reverse total shoulder arthroplasty?

**DOI:** 10.1016/j.jseint.2025.04.025

**Published:** 2025-05-21

**Authors:** Itaru Kawashima, Eric R. Wagner, Joseph J. King, Zaamin B. Hussain, Sameer R. Khawaja, Jaden C. Hadrick, Krishna N. Chopra, Michael B. Gottschalk, Scott A. Banks, Thomas W. Wright

**Affiliations:** a Department of Orthopaedic Surgery and Sports Medicine, University of Florida, Gainesville, FL, USA; b Department of Orthopaedic Surgery, Emory University, Atlanta, GA, USA; c Department of Mechanical & Aerospace Engineering, University of Florida, Gainesville, FL, USA

**Keywords:** Reverse total shoulder arthroplasty, Arthroplasty, Shoulder, Scapulohumeral rhythm, SHR, Kinematics, Preoperative, Postoperative

## Abstract

**Background:**

Reverse total shoulder arthroplasty (rTSA) is believed to alter shoulder kinematics. However, it remains unclear whether the kinematic changes observed in shoulders following rTSA were present preoperatively or developed as a result of the surgery. Furthermore, the impact of preoperative scapulohumeral rhythm (SHR) on the postoperative SHR after rTSA is also poorly understood. The primary aim of this study was to compare shoulder kinematics before and after rTSA using the 3-dimensional (3D) to 2-dimensional model-image registration with dynamic digital radiography images. The secondary purpose was to evaluate whether preoperative SHR correlates with postoperative SHR after rTSA.

**Methods:**

Twenty shoulders in 19 patients that underwent rTSA were included. Dynamic digital radiography images were performed preoperatively and 6 months or later postoperatively to assess shoulder motion during scapular plane abduction. 3D surface models of preoperative scapula and humerus, and postoperative scapula and humerus with implants were created from the preoperative and postoperative computed tomography images. Scapular and humeral kinematics were evaluated using a 3D to 2-dimensional model image registration method. Each kinematics parameter was calculated at 10° increments from 20° to 90° of humeral abduction. The mean SHR was also calculated from the humeral position at side to 90° or to the maximum humeral abduction if it was less than 90°. Linear mixed-effects models for repeated measures were used to compare kinematics at each humeral abduction between preoperative and postoperative conditions. The correlation between the preoperative mean SHR and the postoperative mean SHR was assessed using Pearson's product-moment correlation statistic.

**Results:**

A significant postoperative increase in scapular posterior tilt was observed (*P* = .019). However, changes in SHR, scapular upward rotation, or scapular and humeral internal/external rotation from preoperative to postoperative conditions were not statistically significant. Additionally, a significant correlation was not detected between the preoperative and postoperative mean SHR.

**Conclusion:**

rTSA significantly increases scapular posterior tilt postoperatively compared to the preoperative condition. However, significant changes from the preoperative to postoperative conditions were not demonstrated for other kinematic parameters.

Reverse total shoulder arthroplasty (rTSA) is a nonanatomic procedure designed to restore function to the shoulder through enhanced deltoid muscle utilization.^6^^,^^20^ Due to its nonanatomic design, rTSA is believed to alter shoulder kinematics. In shoulders following rTSA, the scapulohumeral rhythm (SHR) has been reported to be typically low, with motion heavily relying on scapulothoracic movement during abduction.^25^ However, shoulders with massive rotator cuff tears or glenohumeral osteoarthritis have also been reported to exhibit lower SHR.^26^ Therefore, it remains unclear whether the kinematic differences observed in shoulders following rTSA, compared to healthy shoulders, were present preoperatively or developed as a result of the surgery, as well as whether preoperative SHR affects postoperative SHR.

Zaferiou et al^28^ have reported the possibility that SHR may improve postoperatively following rTSA compared to preoperative levels; however, their study included only 12 shoulders, and some showed improvement while others worsened, leaving room for further debate. Furthermore, this study utilized surface markers for analysis, and it appears that no studies have employed the 3-dimensional (3D) to 2-dimensional (2D) model image registration method, which would enable a more direct and precise evaluation of bone or implant movement. Furthermore, it remains unclear how shoulder kinematics such as scapular anterior/posterior tilt and internal/external rotation change before and after rTSA, beyond changes in SHR and scapular upward rotation.

As a method for 3D kinematic analysis of shoulders with rTSA, the 3D-to-2D model image registration approach has been implemented by projecting 3D models created from computed tomography (CT) data onto fluoroscopic images.^25^ However, routine acquisition of fluoroscopic images in clinical practice is challenging. Digital dynamic radiography (DDR) has been reported as a promising, safe, and simple-to-operate technology that can be efficiently and reproducibly integrated into clinical workflows.^26^ To date, the use of DDR images to perform 3D kinematic analysis of shoulders with rTSA using a 3D-to-2D model image registration method has not been commonly reported.

The primary aim of this study was to compare shoulder kinematics before and after rTSA using the 3D-to-2D model image registration method with DDR images. The secondary purpose was to evaluate whether preoperative SHR correlates with postoperative SHR. Our hypothesis was that rTSA would not significantly alter SHR, but based on our clinical observations, it would increase posterior tilting movement compared to the preoperative condition. Furthermore, we hypothesized that preoperative SHR would correlate with postoperative SHR.

## Materials and methods

### Patients

This study was approved by the Institutional Review Board and Ethics Committee of our institution. It included primary rTSA cases for osteoarthritis, cuff tear arthropathy, massive irreparable rotator cuff tear, recurrent instability, or dysplasia, who had DDR and CT images before and after surgery. For the timing of DDR, shoulders that underwent DDR imaging at 6 months or later postoperatively were included, based on a previous study reporting no significant difference in shoulder kinematics during scapular plane abduction between the sixth and 12th postoperative months.^19^ The exclusion criteria included diagnoses of tumors, fractures, fracture sequelae, and shoulders with incomplete or unusable data points.

All surgeries were performed by a fellowship-trained experienced shoulder surgeon at a single institution. Patient records, including surgical reports were reviewed for preoperative demographic information (diagnosis, sex, age at surgery, body weight, height, body mass index, and operative side) as well as implant configuration data. All humeral prostheses utilized a 145° neck–shaft angle. A 36-mm glenosphere was used in 2 shoulders, a 39-mm glenosphere was used in 3 shoulders, a 42-mm glenosphere was used in 2 shoulders, a 36+3-mm glenosphere was used in 6 shoulders, a 36+4-mm glenosphere was used in 3 shoulders, a 39+3-mm glenosphere was used in 1 shoulder, and a 42+3-mm glenosphere was used in 2 shoulders. The thickness of polyethylene inserts was +0 mm in 13 shoulders, +3 mm in 2 shoulders, and +6 mm in 4 shoulders.

### Dynamic digital radiography

**Figure 1:**
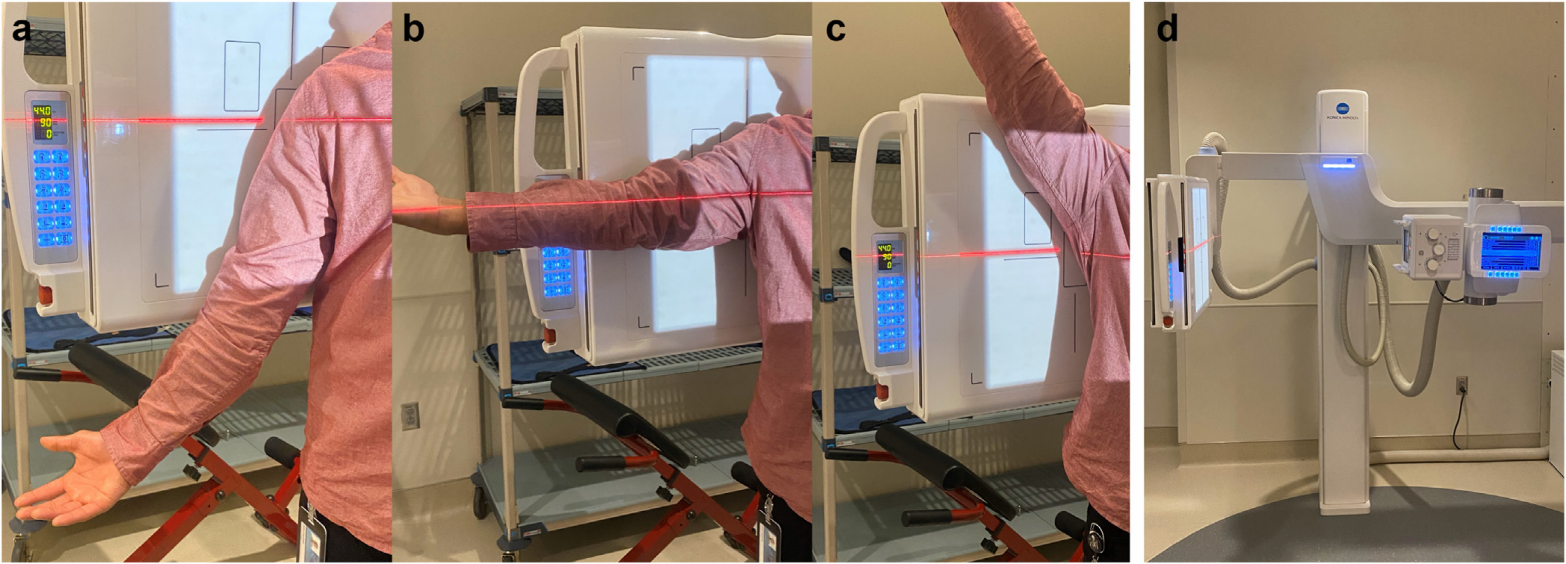
Procedure for capturing DDR images. (**a**-**c**) Patients are positioned in a Grashey view with their posterior shoulder and scapula in contact with the back of the DDR machine. Patients were instructed to stand upright, stationary, avoid axial rotation, and follow a video that provided coordinated instructions on how to perform the motion including standardizing for forearm pronosupination. Patients start with their arm at rest and then gradually abduct their arm to maximum abduction. (**d**) The DDR machine. *DDR*, dynamic digital radiography.

DDR images were taken preoperatively and an average of 12 months postoperatively (range, 6-26), as reported previously (Fig. 1).^7^^,^^8^^,^^11^^,^^12^^,^^26^ In brief, images were obtained as a series of pulsed radiographs at 6-15 Hz for up to 20 seconds. This acquisition creates a series of radiographs during shoulder motion, akin to cineradiography. The specific motion assessed was arm abduction in the scapular plane, starting with the arm at rest by the patient's side and proceeding to maximal abduction. Grashey views were acquired to ensure a view perpendicular to the plane of glenohumeral motion. To standardize image acquisition, the dynamic images were taken under supervision of 1 of 3 licensed radiology technicians, trained using a standardized protocol, who coached patients on timing and cadence during requisite shoulder motions. Patients were instructed to stand upright, stationary, avoid axial rotation, and follow a video that provided coordinated instructions on how to perform the motion including standardizing for forearm pronosupination.

### Three-dimensional implant models

**Figure 2:**
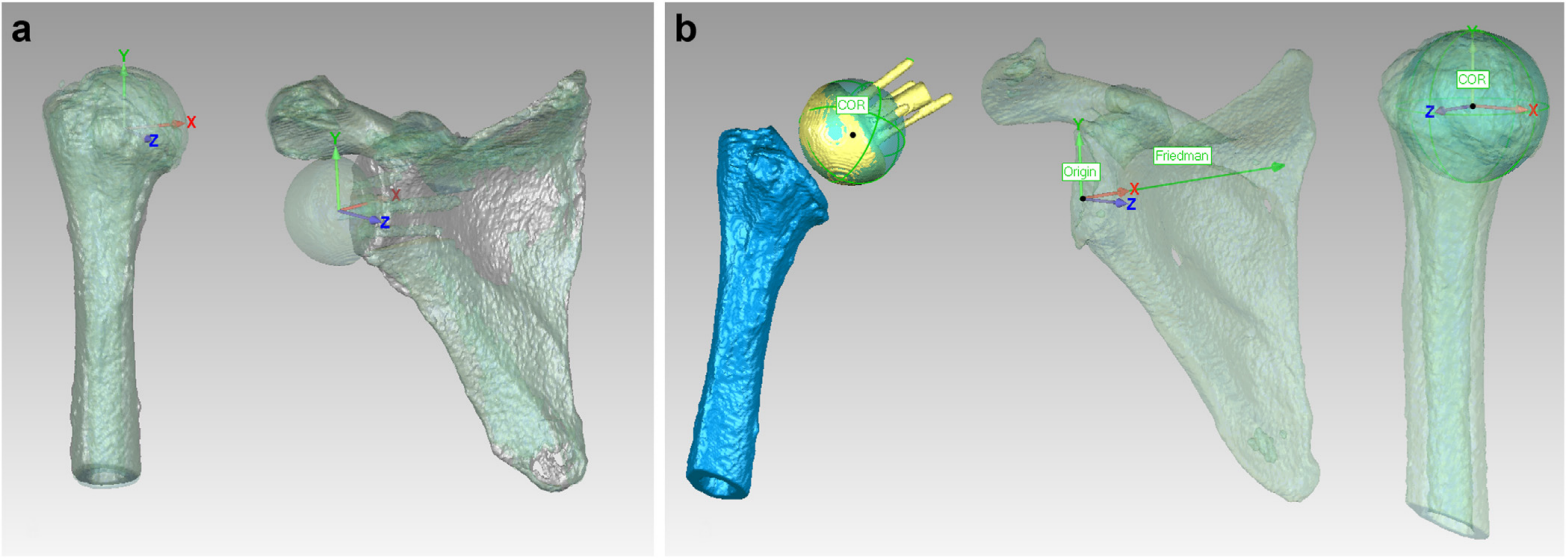
Anatomic coordinate systems of the humerus and the scapula. (**a**) The preoperative and postoperative scapula and humerus were precisely overlaid to align the XYZ axes of the anatomic coordinate system consistently using commercial software (Geomagic Wrap; 3D Systems, Rock Hill, SC, USA). (**b**) The preoperative scapular origin was defined as the midpoint of the line connecting the most superior and inferior bony edges of the glenoid and postoperative scapular origin was set as the center of rotation (COR) of the glenosphere. The preoperative humeral origin was defined as the COR of the humeral head, and the postoperative humeral origin was set at the COR of the glenosphere.

3D surface models of preoperative scapula and humerus, and postoperative scapula and humerus with implants were created from the preoperative and postoperative CT images using segmentation software (ITK snap; Penn Image Computing and Science Laboratory, Philadelphia, PA, USA).^27^ The modeling accuracy has been confirmed by deviation analysis with the root mean square error of 0.4 mm when comparing CT-derived models to the corresponding computer-aided design models.^19^ The preoperative and postoperative scapula and humerus were precisely overlaid to align the XYZ axes of the anatomic coordinate system consistently using commercial software (Geomagic Wrap; 3D Systems, Rock Hill, SC, USA) (Fig. 2, *A*).^16^^,^^19^ The X-axes of all models were set in the mediolateral direction, the Y-axes in the superoinferior direction, and the Z-axes in the anteroposterior direction.^18^^,^^19^ To account for glenoid deformities, the preoperative and postoperative scapular X-axis was aligned using the modified Friedman's line.^3^^,^^4^ The preoperative scapular origin was defined as the midpoint of the line connecting the most superior and inferior bony edges of the glenoid and postoperative scapular origin was set as the center of the glenosphere (Fig. 2, *B*).^16^^,^^18^ The preoperative humeral origin was defined as the center of the humeral head, and the postoperative humeral origin was set at the center of the glenosphere.^16^^,^^18^

### Model image registration and data processing

**Figure 3:**
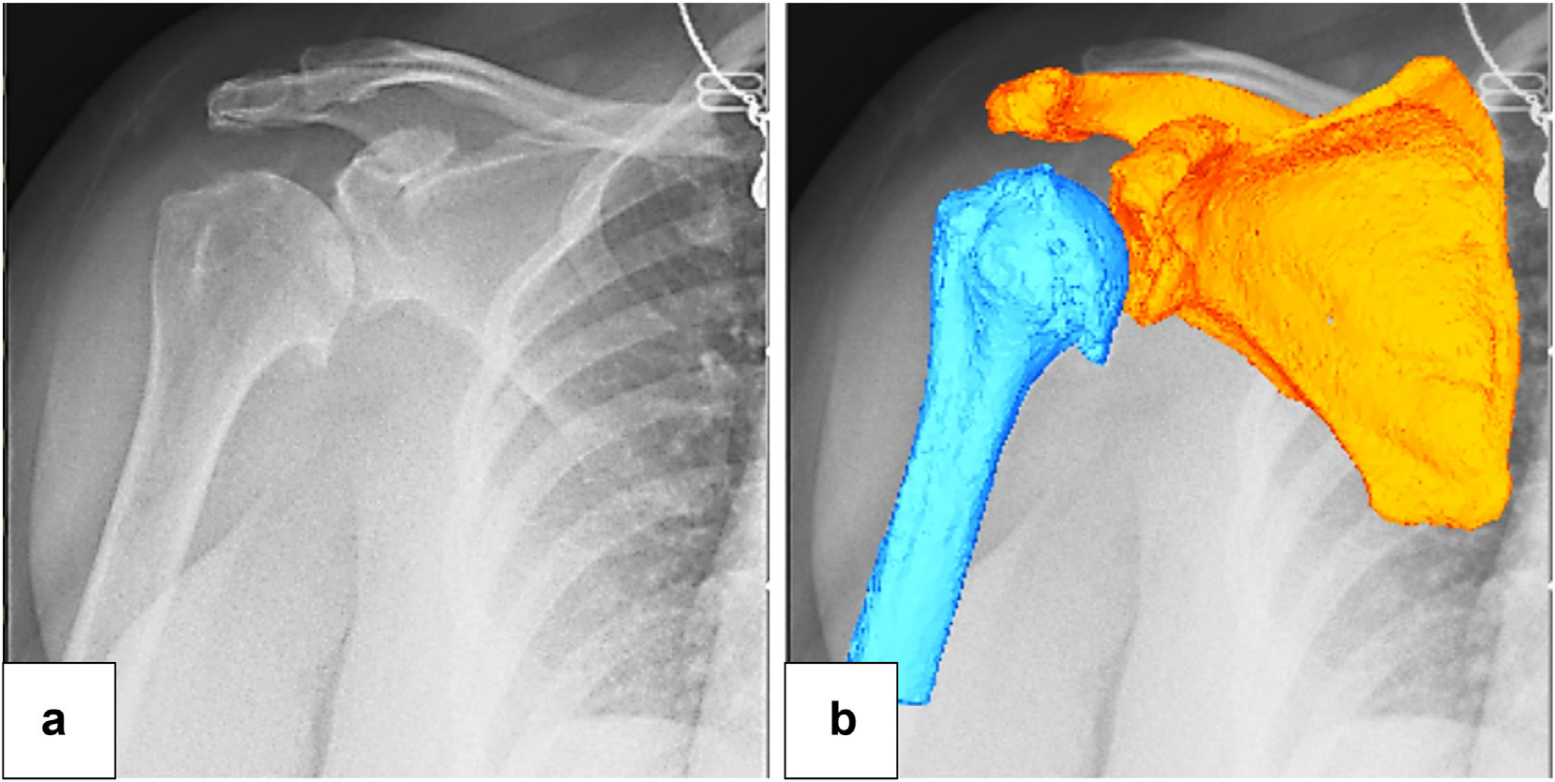
The three-dimensional to 2-dimensional model image registration for reverse total shoulder arthroplasty. (**a**) A DDR image during scapular plane abduction. (**b**) Three-dimensional models were matched with the silhouette of the implants on the DDR image. *DDR*, dynamic digital radiography.

The 3D position and orientation of the scapula and humerus models were determined using model image registration techniques in an open-source software program (JointTrack; University of Florida, Gainesville, FL, USA).^2^^,^^15^ The models were projected onto the DDR image, and their 3D poses were iteratively adjusted to match their silhouettes with the silhouettes in the DDR image (Fig. 3 *A* and *B*). The accuracy of this matching method has been reported to be 0.5 mm and 0.8° for in-plane motions.^17^

The scapula and humerus kinematics relative to the x-ray coordinate system and the kinematics of a humerus relative to a scapula were determined using Cardan angles (Z-X-Y order).^14^ Humeral abduction was defined as rotation about the humeral Z-axis, and internal/external rotation was defined as rotation about the humeral Y-axis. The scapular kinematics were defined as follows: anterior/posterior tilt about X-axis; internal/external rotation about Y-axis; upward/downward rotation about Z-axis.

SHR was defined as (ΔH–ΔS)/ΔS, where ΔH is the increment in humeral abduction angle and ΔS is the increment in scapular upward rotation angle, using a previously reported method.^25^ Each kinematics parameter was calculated at each 10° increment from 20° to 90° of humeral abduction. The mean SHR was also calculated from the humeral position at the side to 90° or to the maximum humeral abduction if it was less than 90°.

### Statistical analysis

All statistical analyses were performed with EZR (Saitama Medical Center, Jichi Medical University, Saitama, Japan), which is a graphical user interface for R (The R Foundation for Statistical Computing, Vienna, Austria).

Linear mixed-effects models for repeated measures were used to compare kinematics at each humeral abduction angle between preoperative and postoperative conditions. The correlation between the preoperative mean SHR and the postoperative mean SHR was assessed using Pearson's product-moment correlation statistic. Preoperative and postoperative mean SHR were compared using unpaired t-tests. *P* values < .05 were considered statistically significant.

## Results

There were 20 shoulders in 19 patients who had CT images before and after surgery and DDR images preoperatively and 6 months or later postoperatively. One shoulder in a patient was excluded due to unusable CT data. Finally, this study evaluated 19 shoulders in 18 patients. The patients consisted of 5 men and 13 women with a mean age of 68 years (range, 42-93) at surgery (Table I). Diagnoses were as follows: osteoarthritis, 9 shoulders; irreparable massive rotator cuff tear, 7 shoulders; cuff tear arthropathy, 1 shoulder; anterior recurrent instability, 1 shoulder; and dysplasia, 1 shoulder (Table II).

**Table I:** Characteristics and implant configuration of included patients.

Shoulder number	Sex	Age at surgery (yr)	Body weight (kg)	BMI (kg/m^2^)	Operative side	Time from surgery to postop-DDR (mo)	Humeral implant system	Glenosphere size (mm)	Poly-insert thickness (mm)
1	Female	72	73.6	26.2	Left	10	Biomet Comprehensive, Onlay	36 + 3	+0
2	Female	66	94.8	37.03	Right	9	Tornier Aequalis Ascend Flex, onlay	36 + 4	+6
3	Female	65	105	38.4	Left	8	Tornier Aequalis Ascend Flex, onlay	36 + 4	+6
4	Female	62	90.9	36.6	Left	12	Tornier Perform, Inlay	36 + 3	+0
5∗	Male	66	120	37	Left	14	Tornier Perform, Inlay	42	+3
6	Female	42	99.8	38.97	Left	8	Tornier Perform, Inlay	36 + 3	+0
7	Female	78	64.1	24.2	Right	13	Tornier Perform, Inlay	39	+0
8	Female	93	90.5	39.6	Left	26	Tornier Aequalis, Inlay	39	+0
9	Female	73	86.6	30.83	Right	12	Tornier Perform, Inlay	36 + 3	+0
10	Female	74	90	35.16	Left	13	Tornier Aequalis Ascend Flex, onlay	39 + 3	+6
11	Male	67	83.2	24.9	Right	22	Tornier Aequalis Ascend Flex, onlay	42 + 3	+6
12∗	Male	65	118	36.3	Right	13	Tornier Perform, Inlay	42	+0
13	Female	60	116.8	41.6	Right	26	Tornier Aequalis, Inlay	36 + 3	+0
14	Female	74	112	43.75	Right	11	Tornier Perform, Inlay	36 + 3	+0
15	Female	78	57	21.56	Left	10	Tornier Aequalis, Inlay	36	+0
16	Male	64	85.3	26.22	Right	6	Tornier Perform, Inlay	42 + 3	+0
17	Female	57	65.3	23.96	Left	6	Tornier Perform, Inlay	36	+0
18	Female	64	95.3	41.01	Right	6	Tornier Perform, Inlay	36 + 4	+3
19	Male	76	66.7	22.35	Left	6	Tornier Perform, Inlay	39	+0

*BMI*, body mass index; *DDR*, dynamic digital radiography.

∗ Shoulder number 5 and 12 belong to the same patient.

**Table II:** Preoperative diagnosis and the mean SHR.

Shoulder number	Preoperative diagnosis	The preoperative mean SHR	The postoperative mean SHR
1	OA	1.51	0.91
2	OA	0.67	0.47
3	OA	0.06	0.33
4	OA	1.71	0.80
5∗	OA	3.28	0.35
6	OA	1.68	1.85
7	OA	1.32	1.20
8	OA	0.51	0.91
9	OA	0.96	1.86
10	MIRCT	0.92	1.00
11	MIRCT	3.65	0.91
12∗	MIRCT	1.75	0.46
13	MIRCT	0.62	0.69
14	MIRCT	3.29	2.09
15	MIRCT	1.94	1.30
16	MIRCT	1.47	0.94
17	CTA	0.48	0.11
18	Anterior RI	0.25	0.31
19	Dysplasia	1.31	1.24

*SHR*, scapulohumeral rhythm; *OA*, osteoarthritis; *CTA*, cuff tear arthropathy; *MIRCT*, massive irreparable rotator cuff tear; *RI*, recurrent instability.

∗ Shoulder number 5 and 12 belong to the same patient.

**Figure 4:**
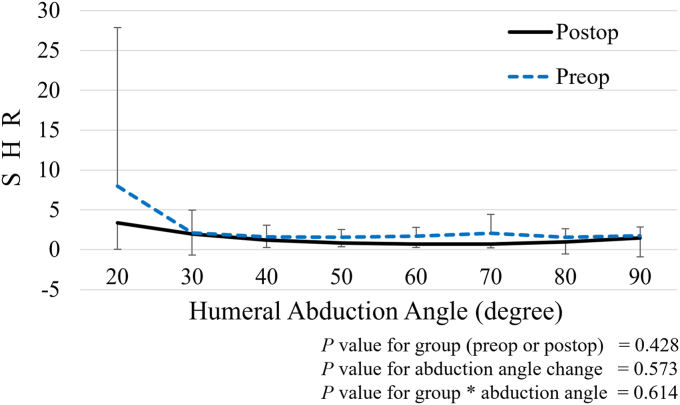
Preoperative and postoperative SHR at each humeral abduction angle. No significant differences or interactions were observed. *SHR*, scapulohumeral rhythm.

**Figure 5:**
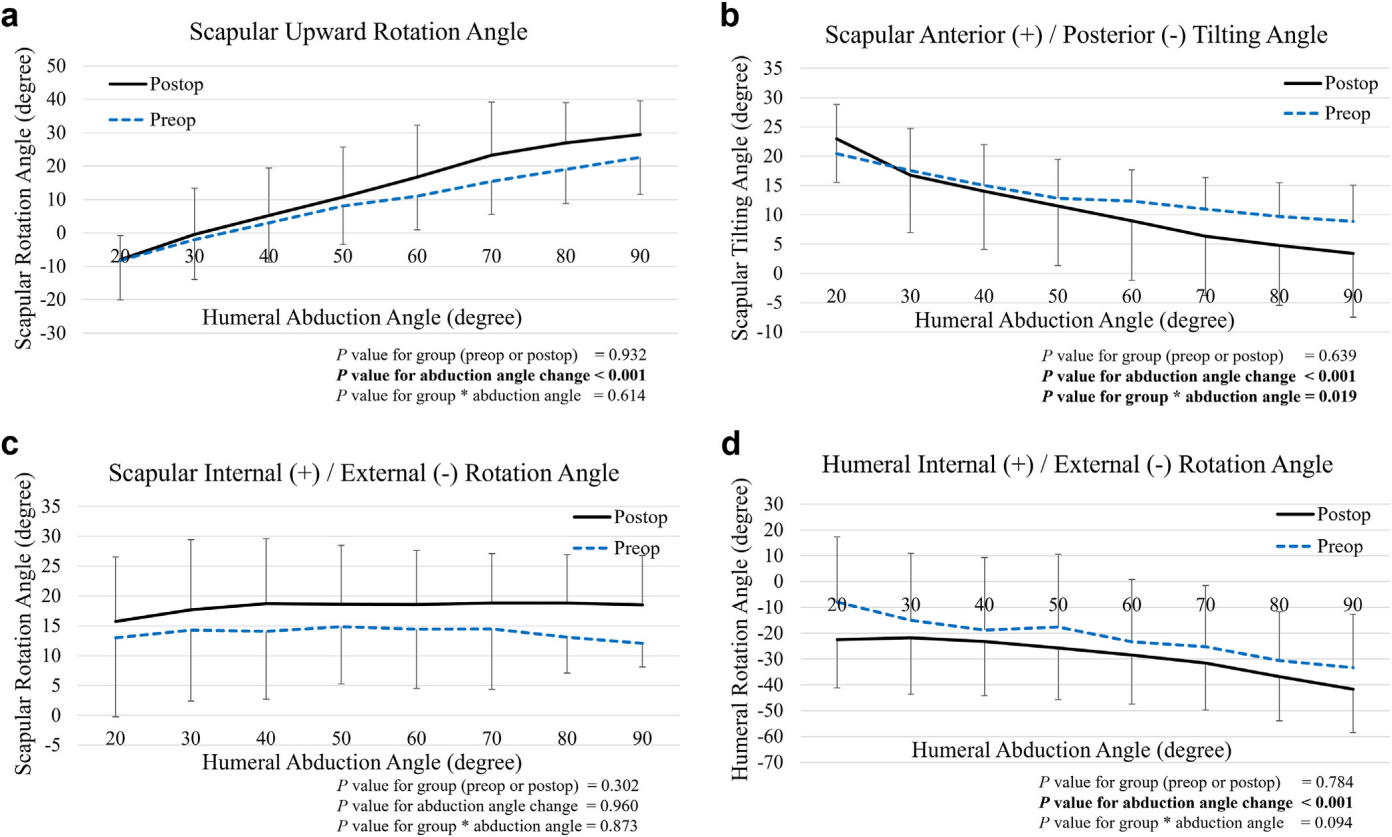
Preoperative and postoperative kinematics at each humeral abduction angle. (**a**) Scapular upward rotation: While it significantly increased with higher humeral abduction angle (*P* < .001), we could not detect a significant difference in this angle between the preoperative and postoperative states. (**b**) Scapular anterior/posterior tilting angle: Scapular posterior tilting angle significantly increased with higher humeral abduction angles (*P* < .001), with a significant interaction effect between group (preoperative or postoperative) and changes in humeral abduction angle (*P* = .019). (**c**) Scapular internal/external rotation angle: No significant differences or interactions were detected. (**d**) Humeral external rotation angle: While this angle significantly increased with higher humeral abduction angles (*P* < .001), no significant difference was detected between preoperative and postoperative states.

There was no significant difference in SHR at each humeral abduction angle between the preoperative and postoperative shoulders (Fig. 4). Although the scapular upward rotation angle was significantly increased when the humeral abduction angle was increased (*P* < .001), there was no significant difference in this angle between preoperative and postoperative states (Fig. 5, *A*). The scapular posterior tilting angle also significantly increased with higher humeral abduction angles (*P* < .001) (Fig. 5, *B*). Additionally, there was a significant interaction effect (group [preoperative or postoperative] ∗ change in humeral abduction angle) on scapular posterior tilting angle (*P* = .019), indicating that the increase in posterior tilting angle was significantly greater in postoperative shoulders compared to preoperative shoulders. The scapular internal/external rotation angle showed no significant difference between preoperative and postoperative shoulders (Fig. 5, *C*). Although the humeral external rotation angle was significantly increased with higher humeral abduction angles (*P* < .001), there was no significant difference in this angle between preoperative and postoperative states (Fig. 5, *D*).

**Figure 6:**
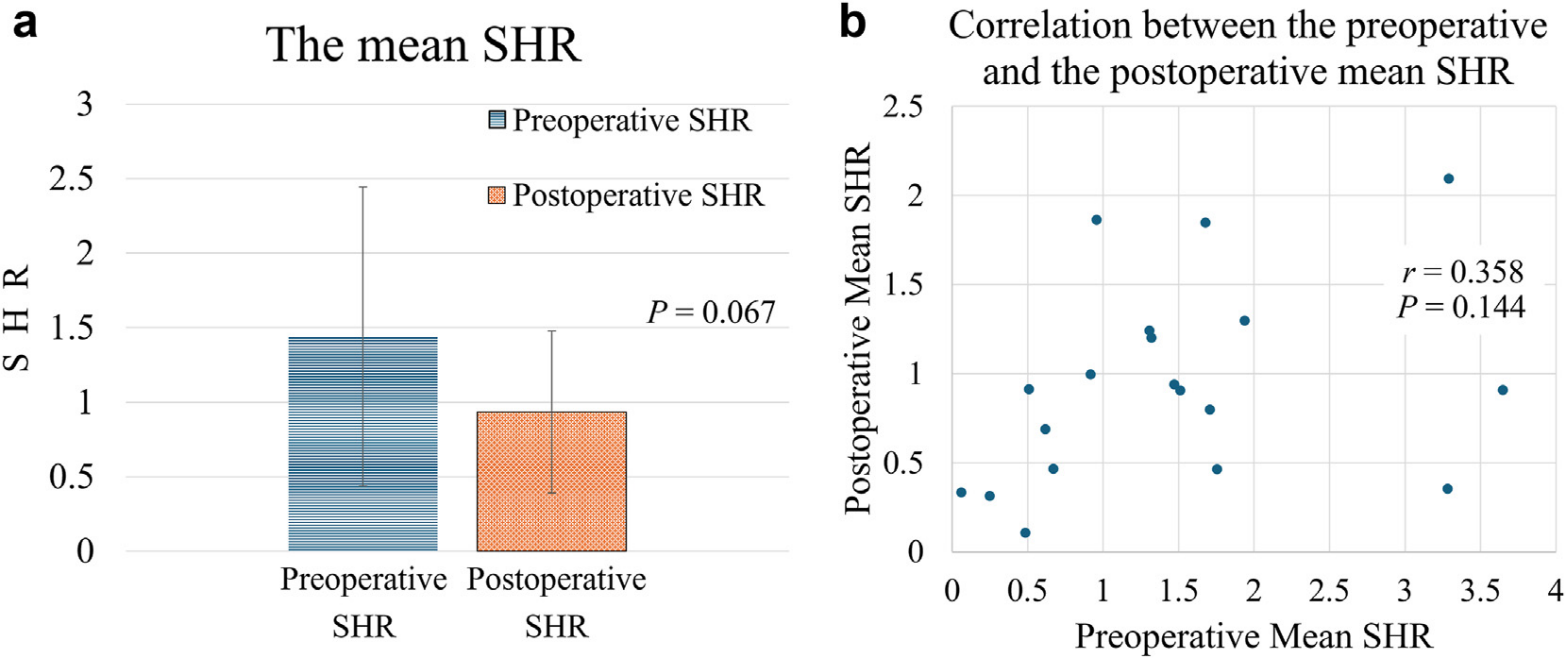
The mean SHR from the humeral position at side to 90° or to the maximum humeral abduction if it was less than 90°. (**a**) No significant difference was detected between the preoperative and postoperative mean SHR. (**b**) There was no significant correlation detected between the preoperative mean SHR and the postoperative mean SHR. *SHR*, scapulohumeral rhythm.

Regarding the mean SHR, the postoperative mean SHR was lower than the preoperative mean SHR in 12 shoulders, while the postoperative mean SHR was higher than the preoperative mean SHR in 7 shoulders (Table II). There was no significant difference between the mean preoperative SHR and the mean postoperative SHR (Fig. 6, *A*). Moreover, there was no significant correlation between the preoperative mean SHR and the postoperative mean SHR (Fig. 6, *B*).

## Discussion

This study, which utilized the 3D-to-2D model image registration with DDR images, demonstrated that shoulders with rTSA increase scapular posterior tilt postoperatively, while significant changes in SHR, scapular upward rotation, or scapular and humeral internal/external rotation from the preoperative to postoperative conditions were not demonstrated. Additionally, a significant correlation between the preoperative and postoperative mean SHR could not be demonstrated.

Several studies have reported that rTSA may alter shoulder kinematics, resulting in decreased glenohumeral motion and compensatory scapulothoracic motion.^13^^,^^23^^,^^25^ However, these findings are based on comparisons between shoulders with rTSA and either healthy or contralateral shoulders.^13^^,^^23^^,^^25^ Interpreting results for shoulders with rTSA vs. healthy shoulders requires caution, as patients undergoing rTSA might already exhibit kinematic differences, including reduced SHR, compared to healthy shoulders.^26^

How the same shoulder changes from pre-rTSA to post-rTSA remained unclear. Zaferiou et al^25^ suggested that SHR may improve postoperatively following rTSA compared to preoperative conditions. However, while the average SHR in their 12 shoulders showed improvement, variability among individual shoulders—some improving and others not—made it challenging to draw definitive conclusions.^28^ Recently, Alexander et al,^1^ in their longitudinal study utilizing a motion capture system with surface markers, demonstrated that SHR exhibited a consistent pattern up to 2 years postoperatively following rTSA, closely resembling the preoperative state. However, their study had a limited number of patients who could be continuously followed up postoperatively, and there was no mention of individual variability. To further investigate, we conducted the present study utilizing the 3D-to-2D model image registration method. In our study there was no statistically significant difference in SHR at each humeral abduction angle between preoperative and postoperative measurements, similar to the result of Alexander et al^1^ Furthermore, similar to the findings of Zaferiou et al,^28^ this study underscores the variability in SHR changes following rTSA (Fig. 5, *A*). While some cases showed improvement from preoperative to postoperative states, others did not, resulting in no significant change in mean SHR when aggregated. A higher postoperative SHR has been reported as beneficial, as it allows for greater elevation or abduction angles.^5^^,^^16^ We hypothesized that the preoperative state might influence postoperative SHR—specifically, that shoulders with higher preoperative SHR would maintain this postoperatively, while those relying more on scapulothoracic motion preoperatively would continue that trend. However, this study did not support this hypothesis, as a significant correlation between the preoperative and postoperative mean SHR could not be demonstrated. Therefore, a low preoperative SHR might not prevent achieving a high SHR postoperatively, nor might a high preoperative SHR guarantee a high SHR after surgery.

Another notable finding in our study was the significant postoperative increase in scapular posterior tilt, which was more pronounced than the increase in upward rotation. In their study comparing muscle activity in rTSA, control, and superior capsular reconstruction (SCR) as surgical options for irreparable rotator cuff tears, Shinagawa et al^22^ reported that serratus anterior muscle activity—associated with scapular posterior tilting movement—was significantly higher in rTSA than in the control and SCR groups. They also found that deltoid activity was significantly elevated in both rTSA and SCR compared to controls, with no notable difference between rTSA and SCR.^23^ The pronounced activation of the serratus anterior, which increases scapular posterior tilting, may be a distinctive feature of rTSA. This increased posterior tilting may help maintain subacromial space, potentially contributing to improved range of motion by reducing impingement in shoulders following rTSA, similar to what is seen in healthy shoulders.^9^ The significant postoperative increase in scapular posterior tilting observed in this study may provide further insight into the kinematic changes that occur after rTSA.

The healthy shoulder demonstrates humeral external rotation during scapular plane abduction, particularly at the early stages of humeral abduction.^18^ This pattern has also been reported in shoulders following rTSA.^24^ In the present study, although no significant changes were observed in scapular internal/external rotation, an increase in humeral external rotation was observed with increasing humeral abduction angles, both preoperatively and postoperatively. However, differences in humeral internal/external rotation between the preoperative and postoperative states could not be demonstrated.

The uniqueness of our present study lies in its ability to capture more direct bone or implant movement, such as scapular posterior tilt, in addition to scapular upward rotation, humeral abduction, and SHR, which could not be measured in previous studies utilizing motion capture systems with surface markers.^1^^,^^28^ Furthermore, this study uses DDR images instead of fluoroscopic images, which have been used in shoulder kinematic analyses with 3D-to-2D model registration methods.^16^, ^17^, ^18^, ^19^^,^^24^^,^^25^ Obtaining fluoroscopic images within the actual clinical workflow is challenging and is typically limited to research or special scenarios. Using DDR images, which can be taken in routine clinical workflows, could facilitate similar research more broadly.^26^ However, the requirement for CT images to create 3D models still presents a barrier, and image-based kinematic analyses have often been time-consuming or cumbersome, restricting their application to research with relatively small cohorts. This issue, however, is being addressed in the field of kinematic analysis after total knee arthroplasty (TKA). For instance, a 3D model of a TKA implant can now be created using only 4 radiographic images,^21^ and machine learning–augmented methods have been employed to develop fully autonomous software pipelines capable of measuring 3D TKA kinematics from single-plane radiographic images.^10^ In the future, the technical issues that currently hinder routine 3D shoulder kinematic measurement may be resolved.

Despite these strengths, our study has limitations. First, the sample size is relatively small, which may limit the generalizability of our findings. Additionally, the relatively small sample size limited us to investigate what factors, including preoperative diagnosis, actually influence kinematics and contribute to improving SHR. As a result, this study focused on the changes in kinematics from preoperative to postoperative conditions. Second, all surgeries were performed by a single experienced shoulder surgeon at one institution, which might introduce a bias in surgical technique and postoperative care. Results could vary with different surgeons and clinical settings. Third, the study used various implant types and configurations, including differences in glenosphere size and polyethylene insert thickness. This variability could introduce inconsistencies in postoperative kinematics. Finally, the heterogeneity of preoperative pathologies or patients' conditions might influence individual kinematic outcomes, making it difficult to isolate the effects solely attributable to rTSA.

## Conclusion

This study, which utilized the 3D-to-2D model image registration with DDR images, demonstrated that rTSA significantly increases scapular posterior tilting postoperatively compared to the preoperative condition. However, other kinematic parameters including SHR, scapular upward rotation, and scapular and humeral internal/external rotation did not demonstrate significant changes from the preoperative to postoperative states. Furthermore, a significant correlation between preoperative and postoperative mean SHR was not identified. This suggests that a low preoperative SHR does not necessarily prevent achieving a high SHR postoperatively, nor does a high preoperative SHR guarantee a high SHR after surgery. These findings should be considered by surgeons when evaluating patients for rTSA and setting postoperative expectations.

## Disclaimers:

Funding: No funding was disclosed by the authors.

Conflicts of interest: Eric R. Wagner is a consultant for Stryker, Smith & Nephew, DePuy Synthes, and Acumed and receives institutional research support from Konica Minolta. Joseph J. King is a consultant for Exactech, Inc. and LinkBio Corp. Michael B. Gottschalk receives institutional support from Skeletal Dynamics, Acumed, and Arthrex. He received research support from Stryker and Konica Minolta. Scott A. Banks is a consultant and receives royalties from Enovis and Stryker. Thomas W. Wright is a consultant and receives royalties from Exactech, Inc. and is a consultant for ABYRX. The other authors, their immediate families, and any research foundations with which they are affiliated have not received any financial payments or other benefits from any commercial entity related to the subject of this article.
